# Unusual Presentation of a Hemiplegic Migraine in a Seven-Year-Old Child: A Case Report

**DOI:** 10.7759/cureus.36726

**Published:** 2023-03-27

**Authors:** Mohamad B Alebaji, Maryam A Ani, Suad A Rozieh, Amar I Al Shibli

**Affiliations:** 1 Department of Academic Affairs, Tawam Hospital, Al Ain, ARE; 2 Department of Pediatric Medicine, Tawam Hospital, Al Ain, ARE

**Keywords:** headache in children, slc4a, renal tubular acidosis type 2, hemi-paresthesia, migraine disorder

## Abstract

In the pediatric population, headache is a common presenting symptom, and migraine is often the diagnosis. A hemiplegic migraine is characterized by an aura and sudden-onset weakness on one side of the body that usually resolves without causing any permanent neurological damage. In this case, we present a seven-year-old male child with a known case of proximal tubular dysfunction (homozygous mutation in the SLC4A4 gene) who presented to the emergency department with a one-day history of weakness on the right side of his body. A few hours after being discharged from the hospital, he began complaining of a severe headache on the left side, accompanied by photophobia, phonophobia, and high fever. Radiology scans and laboratory workup were unremarkable, and encephalitis was ruled out. He was later diagnosed with hemiplegic migraine based on his history and clinical presentation.

## Introduction

Hemiplegic migraine (HM) is a rare form of migraine with aura, as classified by the International Headache Classification [1]. Hemiplegic migraines are uncommon, can be inherited or occur spontaneously, and are associated with a variety of motor, visual, and speech auras [2]. Rare subgroups of individuals may experience more serious symptoms, including temporary or persistent cerebellar ataxia, encephalopathy, or even coma. The presence of motor impairments distinguishes HM from other types of migraine with aura, and its diagnostic criteria have been revised recently (International Classification of Headache Disorders. 3rd Edition (ICHD-3), 2018) [3].

The condition typically appears in the second decade of life, but it can affect anyone between the ages of 1 and 45 with an overall estimated prevalence of 0.01% [4]. It is known that the inherited form is caused by mutations in one of three genes: CACNA1A, ATP1A2, or SCN1A. Recent research suggests that these gene loci are also involved in sporadic disease [5]. According to Moskowitz et al., family mutations increase the brain's susceptibility to extended cortical spreading depression (CSD) caused by either excessive synaptic glutamate release or reduced clearance of glutamate and potassium from the synaptic cleft [6]. In a mouse model, there is a lower threshold for the onset of CSD [7]. Sporadic episodes may present with symptoms such as fever, lethargy, dysphasia, disorientation, hemiparesis, hemisensory complaints, hemianopia, and scintillating scotoma. Often, the symptoms mimic those of a stroke. Thomsen and Olesen recently conducted an evaluation of it [8]. From a pathophysiological standpoint, it is unclear whether sporadic HM represents severe episodes of normal migraine with aura that include motor symptoms in addition to the well-known visual, sensory, and verbal symptoms, or whether it represents a distinct type of migraine with aura. Here, we present a seven-year-old male child with known comorbidities and an unusual manifestation of hemiplegic migraine.

## Case presentation

A seven-year-old male child, a known case of proximal tubular dysfunction (homozygous mutation in the SLC4A4 gene), was admitted to the emergency department with a one-day history of weakness on the right side of his body. The day before, he had undergone superficial keratectomy as a day case and had experienced one episode of vomiting afterward, but he was stable and was discharged home. A few hours after being discharged from the hospital, he began complaining of a severe headache on the left side, accompanied by photophobia and phonophobia. His mother noticed weakness in his right upper and lower limbs, a change in his smile, and slurred speech. All of these symptoms were accompanied by high fever and were unresponsive to antipyretics. He was irritable and unable to recognize his mother.

On physical examination, the child's temperature was 38.7 degrees Celsius, heart rate was 127 beats per minute, respiratory rate was 30 beats per minute, blood pressure was 121/91 mmHg, and oxygen saturation was 99% on room air. His weight and height were in the 29th and 7th percentile, respectively. He was in mild distress and irritable, and hydration was adequate. He was active and alert but slightly disoriented. His pupils were equal, round, and reactive to light, with normal conjunctiva. The rest of his systemic examination was unremarkable.

**Figure 1:**
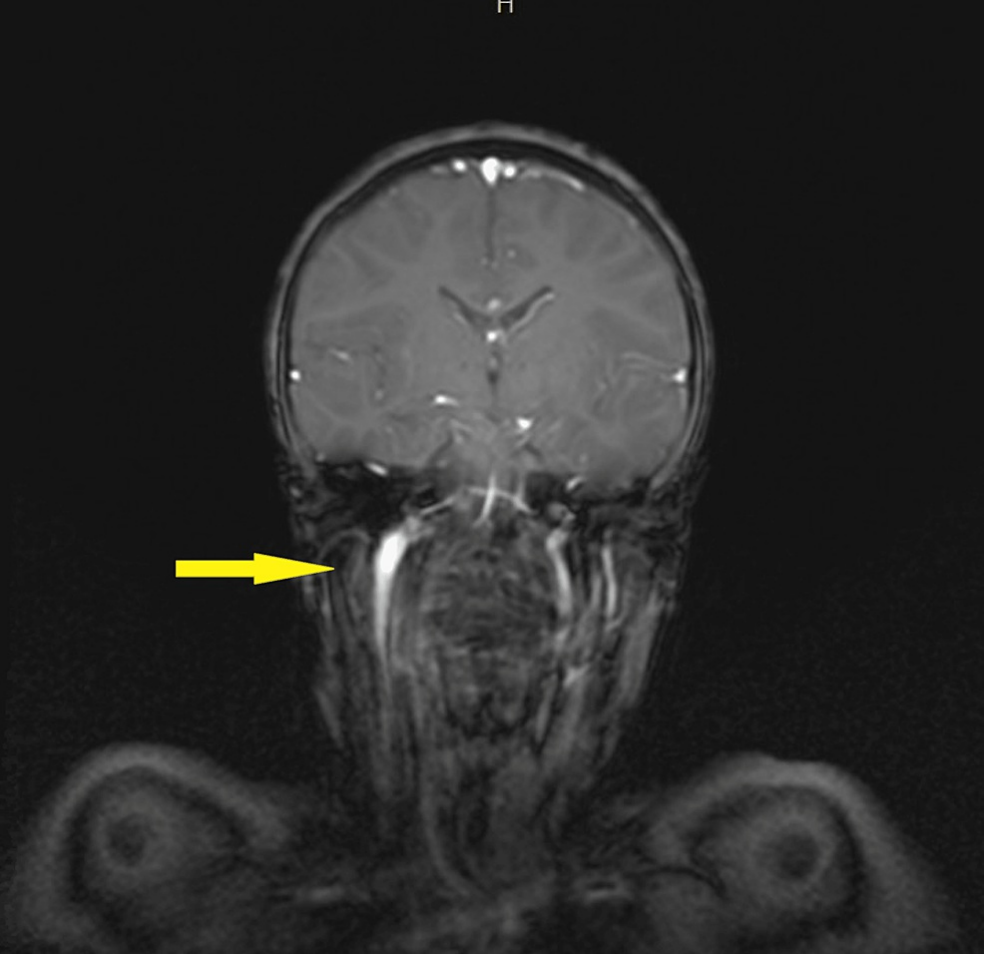
Computed tomography (CT) angiogram showed an impression of relative left peripheral vascularity compared to the right side

Upon arrival, the following lab results were obtained: Na: 150 mEq, K: 3.1 mEq, HCO3: 17, urea: 90 mg/dl, CRP: 0.3 mg/dl, WBC: 11.4x10^9^/L. A chest X-ray showed mild parabronchial cuffing. The computed tomography (CT) angiogram showed an impression of relative left peripheral vascularity compared to the right side (Figure 1).

His CT brain was unremarkable. Magnetic resonance imaging (MRI) of the brain was also unremarkable. To rule out central nervous system (CNS) infection, a lumbar puncture was performed, and the cerebrospinal fluid examination was unremarkable. The child was admitted to the general pediatric department for observation with the impression of a hemiplegic migraine, which could be triggered by emotional stress during the time of anesthesia or by abortive vasoconstrictive drugs (ergotamine and dihydroergotamine). He was stabilized, the pain was managed, and he was started on supportive medications. He responded well and was sent home with safety netting.

## Discussion

Migraine is a complex condition that occurs regularly and is characterized by head pain (usually on one side), vertigo, nausea and vomiting, photophobia, and a dazzling appearance of light. Complicated migraine, of which hemiplegic migraine is a subtype, is defined as the presence of neurologic impairments such as sensory, motor, or speech abnormalities in addition to migraine pain. It was first described by Clark in 1910 [9]. These neurologic abnormalities, known as aura symptoms, typically occur before the headache begins. When the attack ends, the neurologic deficit should resolve completely, although there may be persistent neurologic consequences [10].

The SLC4A4 gene has recently been identified as the source of a condition characterized by metabolic acidosis due to renal failure to acidify urine. This disorder is caused by an abnormal function of the sodium and bicarbonate cotransporter encoded by the gene NBCe1 [11]. Expression of NBCe1 in other organs, such as the CNS, can cause extrarenal symptoms associated with the SLC4A4 mutation. Five out of 15 known SLC4A4 gene mutations affect the CNS and reported occurrences included migraine with or without aura, hemiplegic migraine, episodic ataxia, and epilepsy with stupor in just one case [12]. SLC4A4 is a cause of HM, a rare form of migraine presentation. It is associated with a complex aura that includes, by definition, a motor deficit.

HM is often misdiagnosed as an unusually severe type of migraine, a stroke, multiple sclerosis, metabolic/toxic illness, conversion disorder, or epilepsy (Jacksonian march, Todd's paralysis) at its initial presentation [13]. Clinical diagnosis is also complicated by the clinical and neurobiological similarities between migraine, epilepsy, and HM. Migraine attacks, epilepsy, and HM are thought to be caused by neuronal cortical hyperexcitability [14].

In the past decade, several cases of this condition have been reported. Weng et al. described a 24-year-old male who first presented with generalized weakness that progressed to the right side, along with a pounding headache, right-sided hemianesthesia, hemianopia, and facial palsy [15]. The computed tomography (CT) scan of the brain showed no abnormalities, which was consistent with our case. A diagnosis of familial HM was made based on a history of previous attacks and positive family history. Merwick et al. described a 32-year-old woman with a known familial HM who presented with severe confusion following an HM attack and was initially misdiagnosed as having viral encephalitis [16]. It is quite uncommon in the pediatric population, and the literature is very scarce. However, Ambrosini et al. described a 15-year-old child who experienced recurrent episodes that began with an aura of visual symptoms and unilateral paraesthesias lasting less than 15 minutes and were subsequently identified as hemiplegic migraine [17]. Chen et al. described a three-year-old child with a similar presentation to ours who was diagnosed with HM [18]. Brain imaging scans of most patients are normal. During the attack, diffuse cortical edema may be seen. Eom et al. found a moderately reduced uptake of Diamox 99mTc-HMPAO SPECT in the left frontoparietal vertex in a patient with right hemiplegia [19]. Our patient's scan showed no abnormalities. Pelzer et al. described reversible computed tomography or magnetic resonance imaging abnormalities during and immediately following HM attacks, although lasting computed tomography or magnetic resonance imaging abnormalities are uncommon [20].

## Conclusions

Clinical history and examination are crucial components in diagnosing pediatric neurological diseases. Children with headache problems require a thorough history of the nature and related complaints of the condition in order to rule out uncommon syndromes such as hemiplegic migraine.
